# Trop-2 protein as a therapeutic target: A focused review on Trop-2-based antibody-drug conjugates and their predictive biomarkers

**DOI:** 10.17305/bjbms.2021.6100

**Published:** 2021-06-04

**Authors:** Semir Vranic, Zoran Gatalica

**Affiliations:** 1 Basic Medical Science Department, College of Medicine, QU Health, Qatar University, Doha, Qatar; 2 Biomedical and Pharmaceutical Research Unit, QU Health, Qatar University, Doha, Qatar; 3 Department of Pathology, University of Oklahoma College of Medicine, Oklahoma City, Oklahoma, United States

**Keywords:** Antibody-drug conjugates, breast cancer, predictive biomarkers, sacituzumab govitecan, trophoblast cell-surface antigen-2, urothelial cancer

## Abstract

Antibody-drug conjugates represent a new class of highly potent antineoplastic drugs built by attaching a small molecule of an anticancer drug (payload) or another therapeutic agent to an antibody recognizing an epitope on the targeted cells. Trophoblast cell-surface antigen-2 (Trop-2) was originally described in trophoblasts and fetal tissues, but subsequently its overexpression has been demonstrated in various solid malignancies. Sacituzumab govitecan (SG), a conjugate of anti-Trop-2 antibody and SN-38 payload (an active metabolite of irinotecan), is the first in the class that has been clinically validated and approved by the Food and Drug Administration for the treatment of metastatic triple-negative breast (2020) and urothelial carcinomas (2021). In the current review, we summarize and critically appraise the most recent advances with regard to SG, emphasizing the predictive biomarker analysis.

## INTRODUCTION

### Trophoblast cell-surface antigen-2 (Trop-2) protein as a target for antibody-drug conjugates (ADC)

Trop-2 (also called epithelial glycoprotein-1, gastrointestinal antigen 733-1, membrane component surface marker-1, and tumor-associated calcium signal transducer-2), is a product of the TACSTD2 gene located at 1p32.1 reviewed in Goldenberg et al. [1]. Trop-2 is a 40-kDa glycoprotein that was the first described transducer of intracellular calcium signaling [2,3]. It contains a 274-amino-acid extracellular epidermal growth factor-like repeat portion with three domains, a cysteine-rich domain, a thyroglobulin type-1 domain, and a cysteine-poor domain [1]. The Trop-2 protein interacts with multiple cellular regulators, including Insulin-like growth factor 1, claudin-1, claudin-7, cyclin D1, and Protein kinase C [1]. In addition, various transcription factors closely interact with the TACSTD2 gene, including HNF4A, TP63/TP53, WT1, ERG, HNF1A/TCF-1, and FOXP3 [4,5].

**FIGURE 1:**
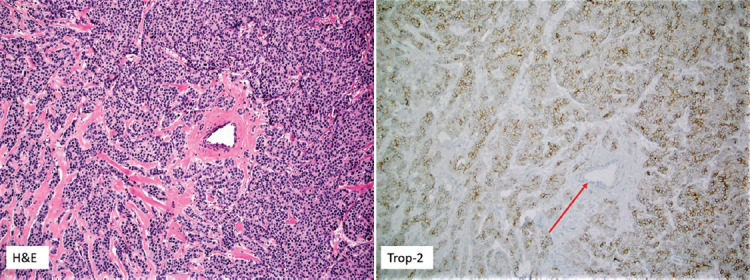
A case of neuroendocrine carcinoma of the breast with trophoblast cell-surface antigen-2 (Trop-2) protein expression (antibody clone Anti-human Trop-2, R and D Systems). Note the membranous expression of Trop-2 protein in cancer cells; the normal breast duct (in the mid part of the figure, red arrow) lacks Trop-2 expression (10× magnification). This case was previously reported in the study by Vranic et al. [14].

Trop-2 expression was originally described in trophoblasts (placenta) and fetal tissues (e.g. lungs). Its expression was subsequently described in the normal stratified squamous epithelium of the skin, uterine cervix, esophagus, and tonsil crypts [6]. However, many normal tissues lack or show low Trop-2 protein expression (e.g. colon, kidney, liver, lung, prostate, and breast) [1]. Figure 1 shows the lack of Trop-2 expression in normal ductal epithelium whereas neoplastic breast cells strongly overexpress Trop-2.

Aberrant Trop-2 overexpression has been described in various solid cancers, including those with low Trop-2 expression in their normal counterparts (e.g. colorectal, renal, lung, and breast carcinomas) (reviewed in Goldenberg et al. [1] and Shvartsur and Bonavida [7]). Trop-2 plays a role in tumor progression, given its active interplay with several key molecular pathways traditionally associated with cancer development and progression [1]. High Trop-2 expression usually confers a poor outcome [8]. In a meta-analysis by Zeng et al. that included 2569 cancer patients (reflecting 13 common solid malignancies), increased Trop-2 expression was particularly associated with poor overall survival (OS) and disease-free survival outcomes in patients with gastrointestinal and gynecological malignancies [8]. Despite the marked limitations of this study (inconsistency in Trop-2 assessment and definition of Trop-2 positivity), the authors concluded that a frequent Trop-2 expression in the majority of solid tumors and its association with a poor prognosis provided a good rationale to target Trop-2 for therapeutic purposes [8]. The Trop-2 expression has also been described in some rare and aggressive malignancies, such as salivary duct carcinomas [9], anaplastic thyroid carcinomas [10], uterine/ovarian carcinosarcomas [11,12], and neuroendocrine carcinoma (NEC) of the prostate [13]. In prostate NEC, Trop-2 appears to closely interplay with the Poly ADP-ribose polymerase enzyme promoting neuroendocrine phenotype and aggressiveness of prostate cancer [13]. We recently reported Trop-2 in ~20% of mammary NEC (Figure 1) [14]. In contrast, our study on cervical NEC revealed marginal (<5% of tested samples) Trop-2 protein expression [15]. Lower Trop-2 expression has also been described in pulmonary and thyroid neuroendocrine neoplasms, which is in contrast to the Trop-2 status in their conventional histotypes (e.g. pulmonary adenocarcinomas and papillary thyroid carcinomas, respectively) [16-18].

## ADC

ADCs represent a new generation of highly potent antineoplastic drugs [19]. These drugs are built by attaching a small molecule of an anticancer drug (payload) or another therapeutic agent to an antibody, using either a permanent or a labile linker. The antibody targets a specific antigen that is preferably over-expressed on malignant cells [20,21]. The linker connects the cytotoxic drug (payload) with the monoclonal antibody and it is responsible for the ADC maintenance and stability in the circulation [21].

Although the first ADCs were synthesized >55 years ago (coupling cyclic chemotherapeutics to immune gamma globulins) [22], their clinical relevance has been limited until 2000 when Gemtuzumab ozogamicin was approved for CD33-positive acute myelogenous leukemia [23]. A decade ago, other ADCs also entered clinical practice, such as Brentuximab vedotin (for Hodgkin lymphoma and anaplastic large cell lymphoma), Trastuzumab emtansine (for HER2-positive breast carcinoma), and Inotuzumab ozogamicin (for B-cell acute lymphoblastic leukemia) among others [20,23-26]. Very recently (December 2019), the Food and Drug Administration (FDA) granted accelerated approval for trastuzumab deruxtecan for the treatment of unresectable or metastatic HER2-positive breast cancer [27]. In addition, enfortumab vedotin was approved for the treatment of locally advanced or metastatic urothelial carcinoma [23] (an overview of selected ADCs approved for the breast and urothelial carcinomas is provided in Table 1).

**TABLE 1:**
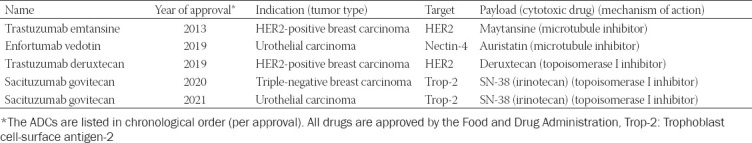
Overview of the antibody-drug conjugates approved for breast (triple-negative and HER2-positive) and urothelial carcinomas. ADCs approved for other indications are not listed

ADCs are a rapidly expanding class of agents with 160 drugs included in preclinical and >70 in clinical trials [20,23]. Nine ADCs have already entered the clinical practice [28]. Two recently approved indications of anti-Trop-2 ADCs are discussed in the following paragraphs.

## ADC USING TROP-2 AS A HOMING TARGET

Two different ADCs targeting the Trop-2 protein have been synthesized, including Sacituzumab govitecan (SG) and RN927C. SG is a conjugate of anti-Trop-2 antibody and SN-38, while in RN927C anti-Trop2 antibody is coupled to a microtubule inhibitor derivate auristatin [1] (Table 1). SG is the first in the class that has been clinically validated and approved by the FDA for heavily pretreated metastatic triple-negative breast and urothelial carcinomas (Table 1). Several other anti-Trop2-based drugs are currently available preclinically, but have not yet entered the clinical trials.

## SG (IMMU-132)

**FIGURE 2:**
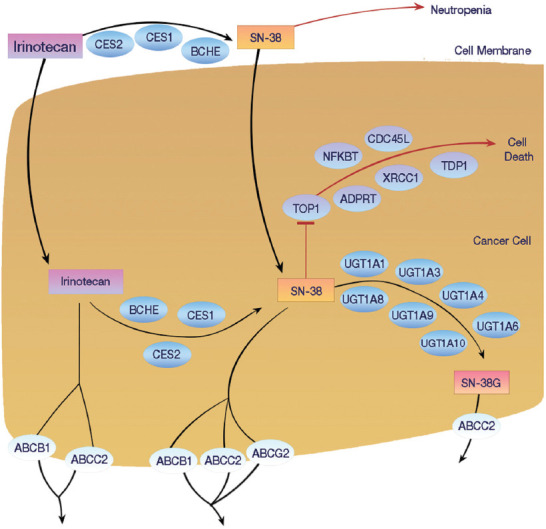
Signaling pathways and genes involved in the irinotecan (and SN-38) metabolism in different compartments of the human body (blood, bile, and intestine) (reproduced from [73] and from the PharmGKB Pathways [74].

SG is a novel, third generation of ADCs [29]. It is composed of a humanized anti-Trop-2 immunoglobulin (Ig)G antibody that is conjugated through a hydrolyzable linker to SN-38, which is an active metabolite of irinotecan [1,30-32]. Irinotecan is a camptothecin that inhibits the nuclear topoisomerase I enzyme, thereby inducing double-stranded DNA breaks during the S-phase of the cell cycle [33] (Figure 2). Enhanced SG uptake by the cancer cells is achieved by the conjugation of a higher number of SN-38 molecules to the Ig. This leads to a drug to antibody ratio = 7-8:1, administration of higher doses (10 mg/kg) and repeated therapy cycles of SG (Days 1 and 8 of 21-day cycles) [33].

## SG (IMMU-132) ACTIVITY IN TRIPLE-NEGATIVE BREAST CANCER (TNBC) AND OTHER SUBTYPES OF BREAST CANCER

In April 2020, the FDA granted accelerated approval to SG (TRODELVY, Immunomedics, Inc.) for the patients with metastatic TNBC that was treated with at least two prior treatment modalities for their metastatic disease [34]. It is the first ADC that has been approved by the FDA for relapsed or refractory metastatic TNBC and is also the first FDA-approved anti-Trop-2 ADC.

The FDA decision was based on the efficacy of SG that had been demonstrated in the IMMU-132-01 (NCT 01631552) clinical trial [35]. NCT 01631552 was a multicenter, single-arm study that enrolled 108 patients with metastatic TNBC. All the enrolled patients received at least two prior therapies for their metastatic disease (median: 3, range, 2–10 therapies). The patients received SG 10 mg/kg intravenously on days 1 and 8 every 21 days. Furthermore, tumor imaging was done every 8 weeks, and patients were treated until disease progression or intolerance to therapy. The two primary outcomes included overall response rate (ORR) and response duration. The ORR was 33.3% (95% CI: 24.6-43.1), while the median response duration was 7.7 months (95% CI: 4.9–10.8) [35]. The recommended SG dose was 10 mg/kg administered by intravenous infusion on days 1 and 8 every 21 days until disease progression or unacceptable toxicity [35]. The most severe side effect reported was myelotoxicity (anemia and neutropenia including febrile neutropenia that affected 9% of the treated patients) [35]. This trial led to the accelerated FDA approval, which was based on ORR and response duration outcomes [34]. Further verification of clinical benefits of SG in TNBC has just been reported in a confirmatory, randomized, phase 3 trials [36]. The trial included 468 patients with metastatic TNBC, excluding brain metastases. The patients were randomly assigned to either SG or classical chemotherapy groups. The objective response rate was significantly higher (35%) in the SG group compared with the chemotherapy group (5%). Consequently, the two groups differed significantly concerning the median progression-free survival (PFS) (5.6 months with SG vs. 1.7 months with chemotherapy) and median OS (12.1 months with SG vs. 6.7 months with chemotherapy). However, myelosuppression and diarrhea were more prevalent in the SG group, the authors reported no deaths directly related to the SG treatment [36].

Trop-2 expression has also been described in estrogen receptor (ER)-positive breast cancers [1,37-39], although it appeared to be lower than in TNBC. This was observed in breast cancer cell lines [40] and in breast tumor samples [14,38,39,41-43] (Figure 1). Recent data also indicate a promising therapeutic activity of SG in patients with luminal (ER+) subtype of breast cancer [44]. Thus, a phase I/II single-arm basket trial involving 54 heavily pretreated patients with ER+/HER2- breast cancer revealed convincing therapeutic effects of SG [44]. At a median follow-up of 11.5 months, the ORR was 31.5%, median duration of response (DOR) was 8.7 months, and median PFS was 5.5 months, while the median OS was 12 months [44]. A new Phase III clinical trial (TROPiCS-02 study, NCT03901339), evaluating SG versus standard treatment in ER+/HER2- metastatic breast cancers, has also been initiated recently and is expected to provide data in the near future [45].

## SG IN UROTHELIAL CARCINOMA

Just a year after the first approval for TNBC, the FDA, in April 2021, has granted another accelerated approval for SG [46]. The drug was approved for patients with locally advanced and/or metastatic urothelial carcinomas who had previously received platinum-containing chemotherapy or immune checkpoint inhibitors (against Programmed cell death receptor/PD-1/or its ligand PD-L1). Efficacy and safety of SG were evaluated in the TROPHY-U-01 trial (IMMU-132-06; NCT03547973) [47]. Patients received SG, 10 mg/kg intravenously, on days 1 and 8 of a 21-day treatment cycle. The main efficacy endpoints were ORR and DOR. ORR and DOR were evaluated by independent review using the response evaluation criteria in solid tumors (RECIST) 1.1 criteria. The confirmed ORR was 27%, with six patients (5%) having complete responses and 25 patients (22%) with partial responses [47]. The median DOR was 7.2 months, while the median OS was 10.9 months. The most common adverse reactions (grade ≥3) in patients receiving SG were neutropenia (35%), leukopenia (18%), diarrhea (10%), and febrile neutropenia (10%). In addition, 6% of the patients had to discontinue the treatment due to severe side effects of SG [47]. Interestingly, this trial included a routine assessment of the Uridine diphosphate glucuronosyltransferase (UGT1A1) gene polymorphisms [47]. Among other functions, this isoform of the UGT1 enzyme glucoronates (inactivates) SN-38 in the liver [48]. However, the presence of the UGT1A1*28 variant that causes reduced UGT activity is associated with the increased risk of irinotecan toxicity. The study confirmed that the presence of UGT1A1*28 was associated with an increased risk of neutropenia in patients with urothelial carcinoma [47].

The recommended SG dose was 10 mg/kg once weekly on days 1 and 8 of 21-day treatment cycles until disease progression or unacceptable toxicity. This indication is approved under accelerated approval based on tumor response rate and DOR. Continued approval of SG for this indication will largely be dependent on the further verification and description of clinical benefits of SG in a confirmatory Phase III trial (3 TROPiCS-04 trial and NCT04527991) [49]. This trial was initiated in November 2020 and is expected to involve approximately 600 patients with advanced/metastatic urothelial carcinomas. No predictive testing is planned for this trial, which is currently in the recruitment phase [49].

## PREDICTIVE BIOMARKERS OF SG EFFICACY

The use of standard molecular marker testing for cancer patient’s helps guide targeted therapy decisions and advances personalized care for these patients. Leading professional bodies (e.g. ASCO, ESMO, and CAP) have been continuously putting efforts into developing and improving the standards of molecular testing in oncology. The ASCO’s recent report on advances in cancer research and treatment further highlighted the importance of biomarker testing (both tissue- and blood-based) in predicting the response, cancer control, side effects, and resistance [50].

There is also emerging evidence indicating that the efficacy of ADCs depends on how their components (antibody, linker, and payload) interact with the cancer cells and tumor microenvironment [28].

Experimental preclinical *in vitro* and *in vivo* data indicate that cell lines that strongly overexpress Trop-2 protein are highly sensitive to SG [51-55]. Further evidence of the experimental efficiency of SG in Trop-2 positive cells was provided in the study by Cardillo et al. [51]. The authors transfected TROP-2 cDNA into the MDA-MB-231 cell line (TNBC cell line), which normally exhibits low Trop-2 protein expression and is unresponsive to SG. However, the transfection resulted in a four-fold increase in Trop-2 expression followed by a significantly higher sensitivity of the breast cancer cells to SG [51].

Phase 1 trial with SG included 25 patients with diverse solid tumors and included predictive immunohistochemistry (IHC) testing for Trop-2 protein; 16 cases had tissue available for Trop-2 IHC, revealing no significant association between the tissue expression of Trop-2 and response to SG [56]. The lack of association in the reported trial could be due to the small sample size. The Phase I/II clinical trial of Bardia et al. [57] that reported the therapeutic benefit of SG in TNBC also included a routine IHC assessment of Trop-2 protein expression with a cutoff of 10%. In contrast to the Phase 1 trial, the authors reported a good association between the IHC expression of Trop-2 and clinical response to SG [57]. These results are in line with preclinical studies. However, two later phase clinical trials on Trop-2 ADC, including the Phase III clinical trials [35,36] did not routinely test the cancer specimens for predictive markers (neither Trop-2 nor Topoisomerase I). This may be acceptable given that TNBC is considered a Trop-2 protein-positive cancer as reported by Goldenberg et al. (~85% positivity rate) [1,37] and 73% in the study of Ambrogi et al. [38]. However, Khoury et al. [58], using the same threshold (10%) for Trop-2 positivity as in Phase I/II trials, reported that only 56% of TNBC were positive for Trop-2 protein. Furthermore, this study found that a substantial discrepancy exists in Trop-2 expression between the primary (49%) and metastatic TNBC (64%) [58]. Intratumoral heterogeneity of Trop-2 expression was also highlighted in the study of Ambrogi et al. [38].

An initial small pilot study (IMMU-132) involving only six heavily pretreated patients with urothelial carcinoma included Trop-2 IHC testing on archival urothelial carcinoma specimens revealed a strong Trop-2 protein expression with a positive correlation to therapy response [59]. A larger, Phase I/II study on urothelial carcinoma (IMMU-132) involved 45 patients with metastatic urothelial carcinoma but did not include predictive biomarker testing [60]. A subsequent TROPHY study did not include the biomarker testing either. Apart from the publicly available data in the Human Protein Atlas [61], very limited information is available concerning the Trop-2 status in urothelial carcinoma. The only two studies available on PubMed/MEDLINE are those of Avellini et al. [62] and Stepan et al. [6]. The first one is based on a small number of invasive urothelial carcinomas (*n* = 10), revealing significantly higher Trop-2 protein expression in invasive carcinomas than non-invasive urothelial carcinomas and normal urothelium [62]. The latter included various normal and cancer tissues of human and mouse origin, indicating a common Trop-2 overexpression in urothelial carcinoma [6].

An active component (payload) of SG is SN-38, which is itself an active metabolite of irinotecan. Irinotecan is a well-known anti-proliferative drug that has been used for the treatment of metastatic colorectal cancer (CRC) [63]. The key molecular target of irinotecan is the topoisomerase I (Topo-1) protein, which belongs to the topoisomerase family of enzymes that are essential in unwinding coiled DNA to facilitate the replication and transcription of the cells [64] (Figure 2). Topo-1 is a nuclear enzyme that is required for replication and unwinding DNA and preventing lethal strand breaks [65,66]. SN-38 is a cytotoxic drug that destabilizes the Topo-1/DNA covalent complex formed in the CRC cells (Figure 2). It induces irreversible double-strand breaks, leading to S-phase arrest, and cell death. This is done by attaching the SN-38 molecule to the complexes and blocks future replication forks preventing repairs of double-strand breaks [66-68].

Topo-1 protein has been found frequently overexpressed in various solid malignancies, including breast cancer [69,70]. A large study by Heestand et al., based on >3,000 breast cancer samples, revealed Topo-1 protein positivity in about 64% of cases [69], while our study in TNBC revealed Topo-1 overexpression in about 70% of the cases [70]. Heestand et al. also reported that 56% of urothelial carcinomas overexpressed Topo-1 expression [69], while our comprehensive theranostic study on urothelial bladder carcinoma reported the Topo-1 positivity rate of 63% [71]. However, the clinical trials failed to confirm a therapeutic benefit of irinotecan alone in patients with advanced/metastatic urothelial carcinomas that were previously treated with one systemic chemotherapy regimen (cisplatin or carboplatin) [72].

Although classical cytotoxic drugs are distinguished from targeted drugs and are generally given to patients without prior biomarker testing, it may be reasonable to further explore the tumor Topo-1 status for its capacity to optimize the response to the drugs such as SG. Our literature survey revealed very limited information in this regard. Cardillo et al. [30] provided solid evidence that SG is more efficient in homologous recombination repair (HRR)-proficient cancer cells with a high Trop-2 expression as well as in the HRR-deficient cancers with low to moderate Trop-2 protein expression [30]. A clinical study of Khoury et al. [58] utilizing the expression of both Trop-2 and Topo-1 in a cohort of primary and metastatic TNBC revealed that ~30% Trop-2-positive cancer were Topo-1 negative. No study (either preclinical or clinical) is currently available regarding the co-expression of Topo-1 and Trop-2 in urothelial carcinoma or other carcinomas. This is an opportunity to develop predictive double biomarker testing for optimization of therapy of complex drugs such as SG and other ADC.

## CONCLUSION

ADCs development and their clinical utility represent one of the most rapidly expanding fields in oncology, with nine currently approved ADCs in clinical practice. In addition, approximately 160 drugs are already included in preclinical and >70 in clinical trials. Although several ADCs, including anti-Trop-2 ADC, have already demonstrated marked therapeutic activity in various hard-to-treat cancers such as metastatic TNBC, HER2-positive, or urothelial carcinomas, more attention should be paid to the identification and development of predictive biomarkers to enhance their efficacy. In addition, a more in-depth and comprehensive understanding of these complex drugs, including the selection of the cell surface targets, antibodies, cytotoxic payload, and the linker technology, will definitely enhance and optimize the efficacy of these promising anticancer agents.

## References

[1] Goldenberg DM, Stein R, Sharkey RM (2018). The emergence of trophoblast cell-surface antigen 2 (TROP-2) as a novel cancer target. Oncotarget.

[2] Lipinski M, Parks DR, Rouse RV, Herzenberg LA (1981). Human trophoblast cell-surface antigens defined by monoclonal antibodies. Proc Natl Acad Sci USA.

[3] Ripani E, Sacchetti A, Corda D, Alberti S (1998). Human trop-2 is a tumor-associated calcium signal transducer. Int J Cancer.

[4] Zaman S, Jadid H, Denson AC, Gray JE (2019). Targeting trop-2 in solid tumors:Future prospects. Onco Targets Ther.

[5] Guerra E, Trerotola M, Aloisi AL, Tripaldi R, Vacca G, La Sorda R (2013). The trop-2 signalling network in cancer growth. Oncogene.

[6] Stepan LP, Trueblood ES, Hale K, Babcook J, Borges L, Sutherland CL (2011). Expression of Trop2 cell surface glycoprotein in normal and tumor tissues:Potential implications as a cancer therapeutic target. J Histochem Cytochem.

[7] Shvartsur A, Bonavida B (2015). Trop2 and its overexpression in cancers:Regulation and clinical/therapeutic implications. Genes Cancer.

[8] Zeng P, Chen MB, Zhou LN, Tang M, Liu CY, Lu PH (2016). Impact of TROP2 expression on prognosis in solid tumors:A systematic review and meta-analysis. Sci Rep.

[9] Wolber P, Nachtsheim L, Hoffmann F, Klussmann JP, Meyer M, von Eggeling F (2021). Trophoblast cell surface antigen 2 (Trop-2) protein is highly expressed in salivary gland carcinomas and represents a potential therapeutic target. Head Neck Pathol.

[10] Seok JY, Astvatsaturyan K, Peralta-Venturina M, Lai J, Fan X (2021). TROP-2, 5hmC, and IDH1 expression in anaplastic thyroid carcinoma. Int J Surg Pathol.

[11] Lopez S, Perrone E, Bellone S, Bonazzoli E, Zeybek B, Han C (2020). Preclinical activity of sacituzumab govitecan (IMMU-132) in uterine and ovarian carcinosarcomas. Oncotarget.

[12] Raji R, Guzzo F, Carrara L, Varughese J, Cocco E, Bellone S (2011). Uterine and ovarian carcinosarcomas overexpressing Trop-2 are sensitive to hRS7, a humanized anti-Trop-2 antibody. J Exp Clin Cancer Res.

[13] Hsu EC, Rice MA, Bermudez A, Marques FJ, Aslan M, Liu S (2020). Trop2 is a driver of metastatic prostate cancer with neuroendocrine phenotype via PARP1. Proc Natl Acad Sci USA.

[14] Vranic S, Palazzo J, Sanati S, Florento E, Contreras E, Xiu J (2019). Potential novel therapy targets in neuroendocrine carcinomas of the breast. Clin Breast Cancer.

[15] Cimic A, Vranic S, Arguello D, Contreras E, Gatalica Z, Swensen J (2021). Molecular profiling reveals limited targetable biomarkers in neuroendocrine carcinoma of the cervix. Appl Immunohistochem Mol Morphol.

[16] Sadullahoglu C, Sayiner A, Suren D, Yildirim HT, Nergiz D, Sezer C (2019). The diagnostic significance of trophoblast cell-surface antigen-2 expression in benign and malignant thyroid lesions. Indian J Pathol Microbiol.

[17] Inamura K, Yokouchi Y, Kobayashi M, Ninomiya H, Sakakibara R, Subat S (2017). Association of tumor TROP2 expression with prognosis varies among lung cancer subtypes. Oncotarget.

[18] Simms A, Jacob RP, Cohen C, Siddiqui MT (2016). TROP-2 expression in papillary thyroid carcinoma:Potential diagnostic utility. Diagn Cytopathol.

[19] Criscitiello C, Morganti S, Curigliano G (2021). Antibody-drug conjugates in solid tumors:A look into novel targets. J Hematol Oncol.

[20] Nejadmoghaddam MR, Minai-Tehrani A, Ghahremanzadeh R, Mahmoudi M, Dinarvand R, Zarnani AH (2019). Antibody-drug conjugates:Possibilities and challenges. Avicenna J Med Biotechnol.

[21] Chau CH, Steeg PS, Figg WD (2019). Antibody-drug conjugates for cancer. Lancet.

[22] Decarvalho S, Rand HJ, Lewis A (1964). Coupling of cyclic chemotherapeutic compounds to immune gamma-globulins. Nature.

[23] Boni V, Sharma MR, Patnaik A (2020). The resurgence of antibody drug conjugates in cancer therapeutics:Novel targets and payloads. Am Soc Clin Oncol Educ Book.

[24] Vranic S, Beslija S, Gatalica Z (2021). Targeting HER2 expression in cancer:New drugs and new indications. Bosn J Basic Med Sci.

[25] Berger GK, McBride A, Lawson S, Royball K, Yun S, Gee K (2017). Brentuximab vedotin for treatment of non-Hodgkin lymphomas:A systematic review. Crit Rev Oncol Hematol.

[26] Wynne J, Wright D, Stock W (2019). Inotuzumab:From preclinical development to success in B-cell acute lymphoblastic leukemia. Blood Adv.

[27] (2019). FDA Approves Fam-Trastuzumab Deruxtecan-Nxki for Unresectable or Metastatic HER2-Positive Breast Cancer.

[28] Drago JZ, Modi S, Chandarlapaty S (2021). Unlocking the potential of antibody-drug conjugates for cancer therapy. Nat Rev Clin Oncol.

[29] Goldenberg DM, Sharkey RM (2020). Sacituzumab govitecan, a novel, third-generation, antibody-drug conjugate (ADC) for cancer therapy. Expert Opin Biol Ther.

[30] Cardillo TM, Rossi DL, Zalath MB, Liu D, Arrojo R, Sharkey RM (2020). Predictive biomarkers for sacituzumab govitecan efficacy in Trop-2-expressing triple-negative breast cancer. Oncotarget.

[31] Vidula N, Ellisen LW, Bardia A (2020). Novel agents for metastatic triple-negative breast cancer:Finding the positive in the negative. J Natl Compr Canc Netw.

[32] Weiss J, Glode A, Messersmith WA, Diamond J (2019). Sacituzumab govitecan:Breakthrough targeted therapy for triple-negative breast cancer. Expert Rev Anticancer Ther.

[33] Goldenberg DM, Sharkey RM (2019). Antibody-drug conjugates targeting TROP-2 and incorporating SN-38:A case study of anti-TROP-2 sacituzumab govitecan. MAbs.

[34] (2020). FDA Grants Accelerated Approval to Sacituzumab Govitecan-Hziy for Metastatic Triple Negative Breast Cancer.

[35] Bardia A, Mayer IA, Vahdat LT, Tolaney SM, Isakoff SJ, Diamond JR (2019). Sacituzumab govitecan-hziy in refractory metastatic triple-negative breast cancer. N Engl J Med.

[36] Bardia A, Hurvitz SA, Tolaney SM, Loirat D, Punie K, Oliveira M (2021). Sacituzumab govitecan in metastatic triple-negative breast cancer. N Engl J Med.

[37] Goldenberg DM, Cardillo TM, Govindan SV, Rossi EA, Sharkey RM (2015). Trop-2 is a novel target for solid cancer therapy with sacituzumab govitecan (IMMU-132), an antibody-drug conjugate (ADC). Oncotarget.

[38] Ambrogi F, Fornili M, Boracchi P, Trerotola M, Relli V, Simeone P (2014). Trop-2 is a determinant of breast cancer survival. PLoS One.

[39] Lin H, Huang JF, Qiu JR, Zhang HL, Tang XJ, Li H (2013). Significantly upregulated TACSTD2 and Cyclin D1 correlate with poor prognosis of invasive ductal breast cancer. Exp Mol Pathol.

[40] Huang H, Groth J, Sossey-Alaoui K, Hawthorn L, Beall S, Geradts J (2005). Aberrant expression of novel and previously described cell membrane markers in human breast cancer cell lines and tumors. Clin Cancer Res.

[41] Zhao W, Kuai X, Zhou X, Jia L, Wang J, Yang X (2018). Trop-2 is a potential biomarker for the promotion of EMT in human breast cancer. Oncol Rep.

[42] Mojica WD, Brandwein-Weber M, Korangy EA (2017). A case of metastatic lobular carcinoma with overexpression of Trop-2:Implications for the consideration of novel therapeutics. Breast J.

[43] Zimmers SM, Browne EP, Williams KE, Jawale RM, Otis CN, Schneider SS (2018). TROP2 methylation and expression in tamoxifen-resistant breast cancer. Cancer Cell Int.

[44] Kalinsky K, Diamond JR, Vahdat LT, Tolaney SM, Juric D, O'Shaughnessy J (2020). Sacituzumab govitecan in previously treated hormone receptor-positive/HER2-negative metastatic breast cancer:Final results from a phase I/II, single-arm, basket trial. Ann Oncol.

[45] Rugo HS, Bardia A, Tolaney SM, Arteaga C, Cortes J, Sohn J (2020). TROPiCS-02:A Phase III study investigating sacituzumab govitecan in the treatment of HR+/HER2-metastatic breast cancer. Future Oncol.

[46] (2021). FDA Grants Accelerated Approval to Sacituzumab Govitecan for Advanced Urothelial Cancer.

[47] Tagawa ST, Balar AV, Petrylak DP, Kalebasty AR, Loriot Y, Flechon A (2021). TROPHY-U-01:A phase II open-label study of sacituzumab govitecan in patients with metastatic urothelial carcinoma progressing after platinum-based chemotherapy and checkpoint inhibitors. J Clin Oncol.

[48] Iyer L, King CD, Whitington PF, Green MD, Roy SK, Tephly TR (1998). Genetic predisposition to the metabolism of irinotecan (CPT-11). Role of uridine diphosphate glucuronosyltransferase isoform 1A1 in the glucuronidation of its active metabolite (SN-38) in human liver microsomes. J Clin Invest.

[49] (2021). Study of Sacituzumab Govitecan (IMMU-132) in Metastatic or Locally Advanced Unresectable Urothelial Cancer (TROPiCS-04).

[50] Smith SM, Wachter K, Burris HA, Schilsky RL, George DJ, Peterson DE (2021). Clinical cancer advances 2021:ASCO's report on progress against cancer. J Clin Oncol.

[51] Cardillo TM, Sharkey RM, Rossi DL, Arrojo R, Mostafa AA, Goldenberg DM (2017). Synthetic lethality exploitation by an anti-trop-2-SN-38 antibody-drug conjugate, IMMU-132, plus PARP inhibitors in BRCA1/2-wild-type triple-negative breast cancer. Clin Cancer Res.

[52] Zeybek B, Manzano A, Bianchi A, Bonazzoli E, Bellone S, Buza N (2020). Cervical carcinomas that overexpress human trophoblast cell-surface marker (Trop-2) are highly sensitive to the antibody-drug conjugate sacituzumab govitecan. Sci Rep.

[53] Perrone E, Manara P, Lopez S, Bellone S, Bonazzoli E, Manzano A (2020). Sacituzumab govitecan, an antibody-drug conjugate targeting trophoblast cell-surface antigen 2, shows cytotoxic activity against poorly differentiated endometrial adenocarcinomas *in vitro* and *in vivo*. Mol Oncol.

[54] Perrone E, Lopez S, Zeybek B, Bellone S, Bonazzoli E, Pelligra S (2020). Preclinical activity of sacituzumab govitecan, an antibody-drug conjugate targeting trophoblast cell-surface antigen 2 (Trop-2) linked to the active metabolite of irinotecan (SN-38), in ovarian cancer. Front Oncol.

[55] Han C, Perrone E, Zeybek B, Bellone S, Tymon-Rosario J, Altwerger G (2020). *In vitro* and *in vivo* activity of sacituzumab govitecan, an antibody-drug conjugate targeting trophoblast cell-surface antigen 2 (Trop-2) in uterine serous carcinoma. Gynecol Oncol.

[56] Starodub AN, Ocean AJ, Shah MA, Guarino MJ, Picozzi VJ, Vahdat LT (2015). First-in-human trial of a novel anti-trop-2 antibody-SN-38 conjugate, sacituzumab govitecan, for the treatment of diverse metastatic solid tumors. Clin Cancer Res.

[57] Bardia A, Mayer IA, Diamond JR, Moroose RL, Isakoff SJ, Starodub AN (2017). Efficacy and safety of anti-Trop-2 antibody drug conjugate sacituzumab govitecan (IMMU-132) in heavily pretreated patients with metastatic triple-negative breast cancer. J Clin Oncol.

[58] Khoury K, Feldman R, Pohlmann PR, Heeke AL, Gatalica Z, Veloso Y (2019). Molecular characterization of trophoblast cell surface antigen 2 (Trop-2) positive triple negative breast cancer (TNBC). J Clin Oncol.

[59] Faltas B, Goldenberg DM, Ocean AJ, Govindan SV, Wilhelm F, Sharkey RM (2016). Sacituzumab govitecan, a novel antibody-drug conjugate, in patients with metastatic platinum-resistant urothelial carcinoma. Clin Genitourin Cancer.

[60] Tagawa ST, Faltas BM, Lam ET, Saylor PJ, Bardia A, Hajdenberg J (2019). Sacituzumab govitecan (IMMU-132) in patients with previously treated metastatic urothelial cancer (mUC):Results from a phase I/II study. J Clini Oncol.

[61] The Human Protein Atlas:TACSTD2.

[62] Avellini C, Licini C, Lazzarini R, Gesuita R, Guerra E, Tossetta G (2017). The trophoblast cell surface antigen 2 and miR-125b axis in urothelial bladder cancer. Oncotarget.

[63] Fujita K, Kubota Y, Ishida H, Sasaki Y (2015). Irinotecan, a key chemotherapeutic drug for metastatic colorectal cancer. World J Gastroenterol.

[64] Cummings J, Smyth JF (1993). DNA topoisomerase I and II as targets for rational design of new anticancer drugs. Ann Oncol.

[65] Paillas S, Causse A, Marzi L, de Medina P, Poirot M, Denis V (2012). MAPK14/p38alpha confers irinotecan resistance to TP53-defective cells by inducing survival autophagy. Autophagy.

[66] Hoskins JM, Rosner GL, Ratain MJ, McLeod HL, Innocenti F (2009). Pharmacodynamic genes do not influence risk of neutropenia in cancer patients treated with moderately high-dose irinotecan. Pharmacogenomics.

[67] Rudolf E, Kralova V, Rudolf K, John S (2013). The role of p38 in irinotecan-induced DNA damage and apoptosis of colon cancer cells. Mutat Res.

[68] Yashiro M, Qiu H, Hasegawa T, Zhang X, Matsuzaki T, Hirakawa K (2011). An EGFR inhibitor enhances the efficacy of SN38, an active metabolite of irinotecan, in SN38-refractory gastric carcinoma cells. Br J Cancer.

[69] Heestand GM, Schwaederle M, Gatalica Z, Arguello D, Kurzrock R (2017). Topoisomerase expression and amplification in solid tumours:Analysis of 24,262 patients. Eur J Cancer.

[70] Millis SZ, Gatalica Z, Winkler J, Vranic S, Kimbrough J, Reddy S (2015). Predictive Biomarker profiling of >6000 breast cancer patients shows heterogeneity in TNBC, with treatment implications. Clin Breast Cancer.

[71] Millis SZ, Bryant D, Basu G, Bender R, Vranic S, Gatalica Z (2015). Molecular profiling of infiltrating urothelial carcinoma of bladder and nonbladder origin. Clin Genitourin Cancer.

[72] Beer TM, Goldman B, Nichols CR, Petrylak DP, Agarwal M, Ryan CW (2008). Southwest oncology group phase II study of irinotecan in patients with advanced transitional cell carcinoma of the urothelium that progressed after platinum-based chemotherapy. Clin Genitourin Cancer.

[73] Whirl-Carrillo M, McDonagh EM, Hebert JM, Gong L, Sangkuhl K, Thorn CF (2012). Pharmacogenomics knowledge for personalized medicine. Clin Pharmacol Ther.

[74] (2021). PharmGKB Pathways.

